# No evidence of the Shiga toxin-producing *E. coli *O104:H4 outbreak strain or enteroaggregative *E. coli *(EAEC) found in cattle faeces in northern Germany, the hotspot of the 2011 HUS outbreak area

**DOI:** 10.1186/1757-4749-3-17

**Published:** 2011-11-03

**Authors:** Lothar H Wieler, Torsten Semmler, Inga Eichhorn, Esther M Antao, Bianca Kinnemann, Lutz Geue, Helge Karch, Sebastian Guenther, Astrid Bethe

**Affiliations:** 1 Institute of Microbiology and Epizootics, Veterinary Faculty, Freie Universität Berlin, Germany; 2 Institute of Epidemiology, Friedrich-Loeffler-Institut, Federal Research Institute for Animal Health, Seestrasse 55, D-16868 Wusterhausen, Germany; 3 Institute for Hygiene and the National Consulting Laboratory for Haemolytic Uraemic Syndrome, University of Münster, Germany

**Keywords:** Shiga toxin, *E.coli*, EAEC, enteroaggregative, O104:H4, HUS, cattle, outbreak

## Abstract

**Background:**

Ruminants, in particular bovines, are the primary reservoir of Shiga toxin-producing *E. coli *(STEC), but whole genome analyses of the current German ESBL-producing O104:H4 outbreak strain of sequence type (ST) 678 showed this strain to be highly similar to enteroaggregative *E. coli *(EAEC). Strains of the EAEC pathotype are basically adapted to the human host. To clarify whether in contrast to this paradigm, the O104:H4 outbreak strain and/or EAEC may also be able to colonize ruminants, we screened a total of 2.000 colonies from faecal samples of 100 cattle from 34 different farms - all located in the HUS outbreak region of Northern Germany - for genes associated with the O104:H4 HUS outbreak strain (*stx2*, *terD*, *rfb*_O104_, *fliC*_H4_), STEC (*stx1*, *stx2*, *escV*), EAEC (*pAA*, *aggR, astA*), and ESBL-production (*bla*_CTX-M_, *bla*_TEM_, *bla*_SHV_).

**Results:**

The faecal samples contained neither the HUS outbreak strain nor any EAEC. As the current outbreak strain belongs to ST678 and displays an en-teroaggregative and ESBL-producing phenotype, we additionally screened selected strains for ST678 as well as the aggregative adhesion pattern in HEp-2 cells. However, we were unable to find any strains belonging to ST678 or showing an aggregative adhesion pattern. A high percentage of animals (28%) shed STEC, corroborating previous knowl-edge and thereby proving the validity of our study. One of the STEC also harboured the LEE pathogenicity island. In addition, eleven animals shed ESBL-producing *E. coli*.

**Conclusions:**

While we are aware of the limitations of our survey, our data support the theory, that, in contrast to other Shiga-toxin producing *E. coli*, cattle are not the reservoir for the O104:H4 outbreak strain or other EAEC, but that the outbreak strain seems to be adapted to humans or might have yet another reservoir, raising new questions about the epidemiology of STEC O104:H4.

## Background

The month of May 2011 marked the beginning of an outbreak of haemolytic uremic syndrome (HUS) caused by an unusual Shiga toxin-producing *E. coli *(STEC) O104:H4 strain, belonging to the HUSEC041 clone (HUS-associated enterohaemorrhagic *E. coli*) because of this specific serotype [1]. The strain, which was found to be of sequence type (ST) 678 rendered many ill and also claimed several lives in Germany. The epicentre of the outbreak was Northern Germany, from where it has spread throughout Germany and beyond, to other European countries [2-4]. With a predominance of infection in adult women and more than 800 cases of haemolytic uremic syndrome (HUS) accompanied with central nervous system complications, this outbreak is unusual. While the reasons for this are currently unknown, it has already been proven that the outbreak strain has an unusual genome make up, as it shows a strong similarity to an enteroaggregative *E. coli *(EAEC) of the same serotype, which was previously isolated from a patient in Africa. Therefore, this *E. coli *strain combines virulence traits of EAEC and STEC [3,5-8].

The principal reservoir of STEC strains are ruminants. However, they have not been reported to harbour STEC O104:H4 nor EAEC [9-11]. To analyse whether the O104:H4 outbreak strain and/or related strains were shed by cattle in the outbreak region, we sought to investigate faecal samples from cattle, originating from the current outbreak area. To address this question, we collected faecal samples from 100 slaughter cattle originating from 34 different farms located in the vicinity of the outbreak area in Northern Germany. Sampling was done on one day in one abattoir. Our findings indicate that the reservoir of the current outbreak strain in fact does not seem to be cattle, addressing the question of whether humans or other so far unrecognized hosts act as a reservoir for these highly pathogenic STEC strains.

## Results

**Figure 1:**
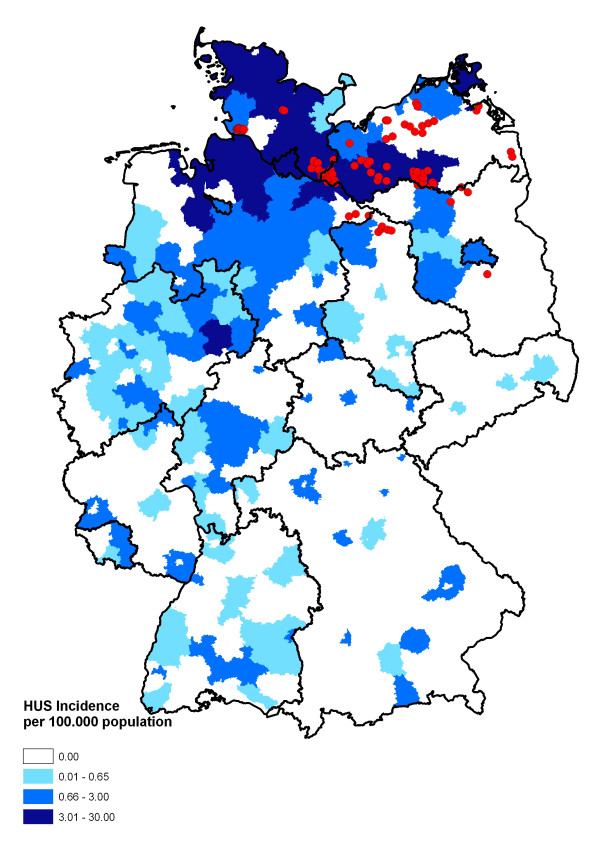
**Map of Germany displaying the incidence of HUS cases (June 29^th^, 2011; source: Robert Koch Institute: SurvStat, http://www3.rki.de/SurvStat)**. Each red dot indicates the origin of a single animal.

On June 6^th^, 2011, during the time of the HUS outbreak, we visited a local abattoir and collected faecal samples of 100 animals, which were slaugh-tered on that day. As shown in Figure 1, most of the 34 farms these animals originated from were located in the outbreak area. The number of animals tested per farm ranged from one to twenty-one (X_med _= 2; X_arith _= 2.94). After cultivation (18 h, 37°C), 20 coliform colonies per animal (2.000 colonies in total) were picked according to the colony morphology and further investigated in a two-step-process: (i) testing the investigated colonies for the presence of the O104:H4 HUS outbreak strain, and (ii) testing the investigated colonies for the presence of STEC and EAEC as well as ESBL-positive strains using both PCR and phenotypic methods.

### Screening bovine faecal *E. coli *for the HUS O104:H4 outbreak strain

In the first part of the present study, the strains were tested using the Multiplex-PCR developed by Bielaszewska et al. (2011) for rapid detection of the outbreak strain. Out of 2.000 *E. coli *isolates derived from the 100 faecal samples investigated, not a single one showed the O104:H4 HUS outbreak strain-specific combination of the genes *stx2*, *terD*, *rfb_O104_*, and *fliC_H4 _*detected by this Multiplex-PCR. Thus, not a single animal shed the O104:H4 outbreak strain.

### Screening bovine faecal *E. coli *for the presence of STEC, EAEC, and ESBL-positive isolates

The second part of the study focussed on the question, whether STEC, EAEC, or ESBL-positive strains are present in the investigated population. For this purpose, the strains were further tested for (i) STEC and EPEC properties, namely *stx1*, *stx2*, *bfpA*, and *escV*; (ii) enteroaggregative *E. coli *(EAEC) properties, especially the occurrence of the genes *pAA*, *aggR*, but also the enteroaggregative adhesion pattern; and (iii) ESBL phenotype.

(i) Twenty-eight of the 100 faecal samples tested harboured STEC. One single sample could be positive for different STEC strains, that is, harbouring isolates either positive for *stx1 *or *stx2 *or both (Table 1). Most of these samples (n = 19) harboured isolates containing *stx2 *only. Eight samples harboured isolates with both *stx1 *and *stx2*, and only one of these samples contained an isolate positive for *stx1 *only. One animal shed an isolate which, in addition to *stx2*, was positive for the *escV *gene, indicating the possession of a Locus of enterocyte effacement (LEE). As for the *bfpA *gene, we were unable to find any positive isolate, thus no typical EPEC were identified.

**Table 1:** Characteristics of STEC identified in the present study (all STEC were ESBL-negative)

Strain	**Animal-no**.	**Farm-no**.	MLST						VAG						Adhesion pattern
			*icd*	*mdh*	*stx2*	*stx1*	*terD*	*rfb_O104_*	*fliC_H4_*	*pAA*	*aggR*	*astA*	*bfpA*	*escV*	
IMT26289	4	F2	16*	12*	+	-	-	-	-	-	-	-	-	-	n.t.
IMT26290	4	F2	16	12	+	-	-	-	-	-	-	-	-	-	n.t.
IMT26294	5	F2	1	20	+	-	-	-	-	-	-	-	-	+	n.t.
IMT26296	16	F6	26	9	+	-	-	-	-	-	-	-	-	-	no defined pattern
IMT26297	21	F11	43	5	+	+	-	-	-	-	-	-	-	-	n.t.
IMT26299	28	F16	1	9	+	+	-	-	-	-	-	-	-	-	LA
IMT26300	28	F16	1	9	+	+	-	-	-	-	-	-	-	-	LA
IMT26303	35	F18	85	7	+	-	-	-	-	-	-	-	-	-	n.t.
IMT26304	37	F18	18	24	+	-	-	-	-	-	-	-	-	-	n.t.
IMT26305	42	F18	26	9	+	+	-	-	-	-	-	-	-	-	no defined pattern
IMT26307	43	F18	109	7	+	-	-	-	-	-	-	-	-	-	n.t.
IMT26308	43	F18	109	7	+	-	-	-	-	-	-	+	-	-	no defined pattern
IMT26309	44	F19	18	24	+	-	-	-	-	-	-	-	-	-	n.t.
IMT26310	46	F19	18	24	+	-	-	-	-	-	-	-	-	-	n.t.
IMT26313	51	F20	26	9	+	+	-	-	-	-	-	-	-	-	no defined pattern
IMT26314	54	F20	16	24	+	-	-	-	-	-	-	-	-	-	n.t.
IMT26315	57	F20	new	new	+	-	-	-	-	-	-	+	-	-	LA
IMT26316	60	F20	new	24	+	-	-	-	-	-	-	-	-	-	no adhesion detected
IMT26317	66	F22	16	12	+	-	-	-	-	-	-	-	-	-	n.t.
IMT26318	68	F22	8	8	+	-	-	-	+	-	-	+	-	-	no defined pattern
IMT26319	71	F24	18	53	+	-	+	-	-	-	-	-	-	-	no defined pattern
IMT26320	73	F25	18	9	+	-	-	-	-	-	-	-	-	-	DA
IMT26321	73	F25	16	7	+	-	-	-	-	-	-	-	-	-	n.t.
IMT26322	74	F26	1	9	+	+	-	-	-	-	-	-	-	-	no defined pattern
IMT26324	74	F26	8	8	+	-	-	-	-	-	-	-	-	-	n.t.
IMT26325	74	F26	18	9	+	-	-	-	-	-	-	-	-	-	no defined pattern
IMT26326	75	F34	16	24	+	-	-	-	-	-	-	-	-	-	n.t.
IMT26327	75	F34	16	24	+	-	-	-	-	-	-	-	-	-	n.t.
IMT26329	75	F34	16	24	+	-	-	-	-	-	-	-	-	-	n.t.
IMT26330	75	F30	16	24	+	-	-	-	-	-	-	-	-	-	no defined pattern
IMT26331	77	F28	103	24	+	-	-	-	-	-	-	-	-	-	n.t.
IMT26332	80	F10	85	7	+	-	-	-	-	-	-	-	-	-	n.t.
IMT26334	80	F10	45	11	-	+	-	-	-	-	-	-	-	-	n.t.
IMT26335	83	F10	26	9	+	+	-	-	-	-	-	-	-	-	no defined pattern
IMT26336	83	F10	85	7	+	-	-	-	-	-	-	-	-	-	n.t.
IMT26337	83	F10	85	7	+	-	-	-	-	-	-	-	-	-	n.t.
IMT26338	83	F10	85	7	+	-	-	-	-	-	-	-	-	-	n.t.
IMT26340	90	F10	16	11	+	-	-	-	-	-	-	-	-	-	n.t.
IMT26341	92	F10	16	11	+	-	-	-	-	-	-	-	-	-	n.t.
IMT26342	92	F10	26	9	+	-	-	-	-	-	-	-	-	-	no defined pattern
IMT26343	93	F10	26	9	+	+	-	-	-	-	-	-	-	-	no defined pattern
IMT26344	93	F10	26	9	+	+	-	-	-	-	-	-	-	-	no defined pattern
IMT26346	93	F10	85	7	+	-	-	-	-	-	-	-	-	-	n.t.
IMT26355	98	F10	103	24	+	-	-	-	-	-	-	-	-	-	n.t.

STEC: Shiga-toxin-producing *E. coli*; ESBL: extended-spectrum beta-lactamase; n.t. not tested; LA: localized adherence; DA: diffuse adherence; *: allele number as assigned by mlst.net

(ii) None of the animals shed any isolates that showed typical genetic features of EAEC, as not a single sample could be identified, that harboured isolates reacting positive for *pAA *or *aggR*. In contrast, a large number of animals shed isolates that reacted positively for *astA*, the gene encoding an enterotoxin, which had originally been identified in EAEC, but is not specific for this pathotype. In summary, while STEC were shed by 28% of the investigated animals, not a single animal shed EAEC.

(iii) Eleven of the one hundred animals tested shed a total of 14 ESBL-producing *E. coli *as identified using the CHROMagar orientation supple-mented with cefotaxime. These animals originated from nine different farms. PCR based screening and subsequent sequence analysis revealed *bla*_CTX-M-1 _(n = 12) as the most common ESBL-resistance determinant. Additionally, we found a single *bla*_TEM_-positive isolate, which was identified as *bla*_TEM52_, and another ESBL-producing isolate positive for *bla*_CTX-M-15_. As outlined in Table 2 some ESBL-producing strains further possessed different virulence factors. The single *bla*_CTX-M-15_-positive strain IMT26356 harboured genes *terD *and *astA*. Also, four of the *bla*_CTX-M1_-positive strains were positive for *fliC_H4_*, the gene encoding H-antigen 4, but none possessed O104-encoding genes.

**Table 2:** Characteristics of ESBL-producing *E.coli *identified in the present study

Strain	**Animal-no**.	**Farm-no**.	ESBL type	MLST						VAG					
				*icd*	*mdh*	*stx2*	*stx1*	*terD*	*rfb_O104_*	*fliC_H4_*	*pAA*	*aggR*	*astA*	*bfpA*	*escV*
IMT26356	7	F4	CTX-M-15	96*	70*	-	-	+	-	-	-	-	+	-	-
IMT26358	12	F7	CTX-M-1	18	11	-	-	-	-	-	-	-	+	-	-
IMT26359	18	F30	CTX-M-1	8	8	-	-	-	-	-	-	-	-	-	-
IMT26360	24	F12	CTX-M-1	1	8	-	-	-	-	-	-	-	-	-	-
IMT26362	31	F17	CTX-M-1	8	8	-	-	-	-	+	-	-	-	-	-
IMT26363	31	F17	CTX-M-1	8	8	-	-	-	-	+	-	-	+	-	-
IMT26364	34	F17	CTX-M-1	8	8	-	-	-	-	-	-	-	+	-	-
IMT26365	35	F18	CTX-M-1	8	8	-	-	-	-	-	-	-	-	-	-
IMT26366	35	F18	CTX-M-1	16	9	-	-	-	-	-	-	-	-	-	-
IMT26367	36	F18	CTX-M-1	8	12	-	-	-	-	+	-	-	-	-	-
IMT26368	36	F18	TEM-52	13	36	-	-	-	-	+	-	-	-	-	-
IMT26369	72	F24	CTX-M-1	1	8	-	-	-	-	+	-	-	-	-	-
IMT26370	76	F27	CTX-M-1	16	12	-	-	-	-	-	-	-	-	-	-
IMT26374	79	F10	CTX-M-1	8	8	-	-	-	-	-	-	-	-	-	-

n.t.: not tested; *: allele number as assigned by mlst.net

### MLST

**Figure 2:**
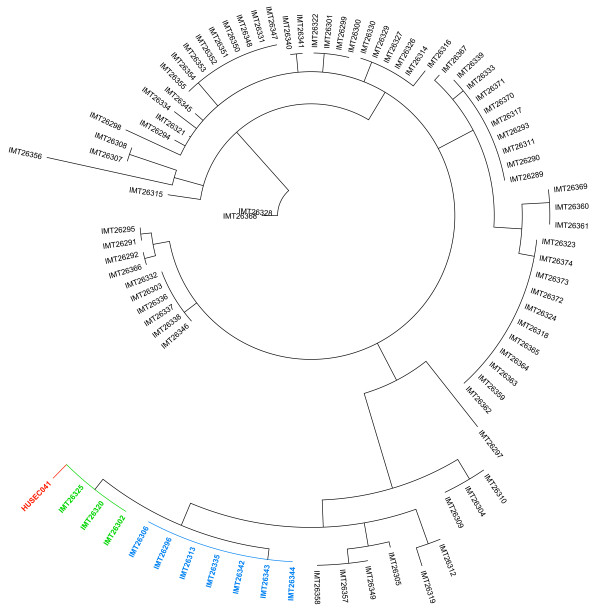
**Dendrogram based on the analyses of *icd *and *mdh *from 86 bovine *E. coli *strains**. Strains highlighted in green (*icd *18) and blue (*icd *26), show the closest relationship to O104:H4 outbreak strain, the ST678 reference strain.

Even though we were unable to identify either the O104:H4 outbreak strain or any EAEC, nevertheless we chose a total of 86 strains with at least one characteristic of the O104:H4 HUS-outbreak strain for further proof of our results. As the phylogenetic relationship of *E. coli *strains is more accurately reflected by its sequence type than by its serotype, we first sequenced the two housekeeping genes *icd *and *mdh*, the alleles of which are able to specifically determine strains of ST678. As the outbreak strain belongs to ST678, the unique combination of *icd *136 and *mdh *9 determines the existence of any phylogenetically related strain. The 86 strains were selected for MLST on the following bases: (i) ESBL-producing strains, (ii) strains positive for *stx2*, (iii) strains positive for *fliC_H4_*, and (iv) strains positive for *terD*. However, we did not find the unique ST678 allele combination of *icd *136 and *mdh *9 in any of these investigated strains. While *icd *allele 136 was never found, 18 strains contained *mdh *allele 9. Of these 18 strains that where potentially related to each other due to the common *mdh *allele, we identified 10 strains to be related to ST678 by Maximum Parsimony analysis (Figure 2). Three of them showed the *icd *allele 18 and seven strains shared the *icd *allele 26. As *icd *18 differs in only one and *icd *26 in just two nucleotides from *icd *136, we additionally performed a complete MLST analyses for these 10 strains to further determine the degree of relatedness of these strains to ST678.

The sequence types identified in these 10 strains differed in around 3 to 5 alleles from ST678 of the O104:H4 outbreak strain. An additionally performed clustering based on the nucleotide differences of the 7 MLST genes further substantiated these results as they clearly identified a rather distant similarity between these 10 strains and the O104:H4 outbreak strain (Table 3 and additional file 1).

**Table 3:** Characteristics of 10 *E.coli *chosen for complete MLST analysis

Strain	**Animal-no**.	**Farm-no**.				MLST								VAG							Adhesion pattern
			ST	*adk*	*fumC*	*gyrB*	*icd*	*mdh*	*purA*	*recA*	*stx2*	*stx1*	*rfb_O104_*	*fliC_H4_*	*terD*	*pAA*	*aggR*	*astA*	*bfpA*	*escV*	
IMT26296	16	F6	297	6	65	32	26	9	8	2	+	-	-	-	-	-	-	-	-	-	No defined pattern
IMT26302	32	F17	2425	6	6	3	18	9	7	6	-	-	-	+	-	-	-	-	-	-	LA
IMT26306	42	F18	718	6	19	3	26	9	8	6	-	-	-	-	+	-	-	-	-	-	No defined pattern
IMT26313	51	F20	297	6	65	32	26	9	8	2	+	+	-	-	-	-	-	-	-	-	No defined pattern
IMT26320	73	F25	442	6	95	33	18	9	8	14	+	-	-	-	-	-	-	-	-	-	DA
IMT26325	74	F26	677	6	95	15	18	9	8	14	+	-	-	-	-	-	-	-	-	-	No defined pattern
IMT26335	83	F10	297	6	65	32	26	9	8	2	+	+	-	-	-	-	-	-	-	-	No defined pattern
IMT26342	92	F10	718	6	19	3	26	9	8	6	+	-	-	-	-	-	-	-	-	-	No defined pattern
IMT26343	93	F10	297	6	65	32	26	9	8	2	+	+	-	-	-	-	-	-	-	-	No defined pattern
IMT26344	93	F10	297	6	65	32	26	9	8	2	+	+	-	-	-	-	-	-	-	-	No defined pattern
HUSEC041	None	none	678	6	6	5	136	9	7	7	+	-	+	+	+	+	+	-	-	-	AA

ST: Sequence Type; LA: localized adherence; DA: diffuse adherence; AA: aggregative adherence

### Testing for the enteroaggregative adhesion pattern

Because it is known that EAEC are a diverse type of pathogens [12], in addition to testing for EAEC specific genes *pAA *or *aggR*, we tested 27 strains with some similarity to the outbreak strain, namely possession of virulence gene combinations similar to that of the outbreak strain and/or showing an ESBL phenotype, and/or a slight relation to ST678 as determined by MLST, in the HEp-2 cell adhesion assay for the enteroaggregative adhesion pattern. The features of these 27 tested strains are given in additional file 2. Not a single strain displayed this adhesion pattern, while EAEC 17-2 and the HUS outbreak strain RKI II-2027 did (data not shown). Therefore, our cumulative data show that the investigated animals did not shed any EAEC.

## Discussion

To the best of our knowledge, this is the first study which actively screened cattle during the O104:H4 ST678 HUS outbreak in Germany by simultaneously taking samples of 100 slaughter cattle from 34 different farms, which were located in close proximity of the outbreak area in Northern Germany in June 2011. By screening for the three characteristics that make the current outbreak strain so unique, namely Shiga toxin 2, serotype O104:H4, and tellurite resistance, utilizing the PCR protocol established by Bielaszewska et al. [3] and phenotypically testing for ESBL-production, we could clearly show that none of the samples harboured the outbreak strain. This suggests that the strain was not shed by any of the animals tested. This result was also supported by additional sequence analyses, namely MLST and further phylogenetic analyses, which could clearly show that none of the strains that were found in the cattle are closely related to the O104:H4 outbreak strain.

In addition, we were not able to detect any strains that belonged to the EAEC pathotype in any of the samples. Furthermore, cell culture tests unravelling the adhesion pattern of strains that shared at least some of the features of the outbreak strain did not reveal any strains with an aggregative adhesion pattern, which due to the large diversity of EAEC is still the gold standard for testing [13].

We are aware that active testing of 100 samples does not give sound risk assessment about the possibility of cattle serving as an infection source. Nevertheless, given the fact that (i) we tested these animals during the HUS outbreak [3,14] and (ii) EAEC have not been found in previous studies in cattle [9-11], our findings indicate that cattle are not the source of this current outbreak. In effect, while we confirm previous findings on cattle not shedding EAEC, our study is important with respect to the fact that ruminants, and mainly cattle, are the primary reservoir of STEC. Therefore, our results, showing that neither the O104:H4 outbreak strain nor any EAEC could be isolated, strongly hint towards the possibility that cattle are not the reservoir of the O104:H4 outbreak strain. Hence, the reservoir of these special STEC is unknown and further work is needed to answer this question.

According to the literature EAEC are highly adapted to humans only, which would suggest the human to be the reservoir [13,15]. This indeed would have a profound impact on infection epidemiology, particularly when taking into account the role of humans who shed the HUS outbreak strain without developing disease. While it is known from previous studies that STEC are shed, it can therefore be openly speculated for how long and how intensely this strain could then be shed.

We were able to isolate STEC strains from 28% of the animals, which also is an expected finding, substantiating our previous work and that of other colleagues from Germany [16-20]. It is however worth mentioning, that all of these animals shed one or more strains positive for *stx2*, while only eight animals shed strains producing *stx1*, all in addition to *stx2*-positive strains.

Another interesting finding was the isolation of 14 ESBL-producing *E. coli *out of 11 animals originating from nine different farms, meaning that 11% of the animals actually shed ESBL-producing *E. coli*, mainly harbouring the gene *bla*_CTX-M-1_. We are not aware of too many studies detecting such strains in faecal samples of cattle, but this high percentage of ESBL-producers is comparable to the so far limited knowledge given by other studies, where up to 39% of the cattle of a single farm were ESBL-producers mostly carrying *bla*_CTX-M-1 _[21-24]. Nearly all ESBL-positive strains we isolated belonged to this group, suggesting that such strains are obviously common in cattle worldwide.

In summary our data indicate that cattle are not the reservoir for the O104:H4 outbreak strain or EAEC. Clearly the identification of the ecology of the O104:H4 strains requires further investigation.

## Conclusions

No O104:H4 outbreak strains could be detected in the investigated cattle, while other STEC were present. In addition, no *E. coli *showing character-istics of closely related *E. coli*, e.g. *aggR*, *pAA *were isolated from the samples, indicating that O104:H4 and EAEC may possibly have a reservoir other than cattle, which is known to be the reservoir for "classical" STEC like serotype O157:H7. This work presents the first step in the process of identifying the host and res-ervoir of the O104:H4 outbreak strain.

## Materials and methods

### Reference strains

The following strains were used as reference strains for all experiments elaborated as described in the Materials and Methods section: Strain HUSEC041 (ST678; O104:H4; [3]) and HUS-outbreak strain RKI II-2027 (ST678; O104:H4, kindly provided by Angelika Fruth and Erhard Tietze, Robert Koch Institute Wernigerode, Germany) were the HUSEC http://www.ehec.org/ and outbreak reference strains, respectively. The STEC reference strain was EDL933 (ST11, O157:H7), while strain 17-2 (ST10; O3:H2) [25] served as EAEC reference strain. In addition, *E. coli *strain E2348/69 (ST15; O127:H6) was used as an EPEC reference strain (Table 4).

**Table 4:** Characteristics of the reference strains used in the present study

Strain	Pathotype	Serotype	ST					VAG				Adhesion pattern	ESBL phenotype
				*stx2*	*terD*	*stx1*	*pAA*	*aggR*	*astA*	*bfpA*	*escV*		
HUSEC041	STEC	O104:H4	ST678	+	+	-	+	+	-	-	-	AA	negative
RKI II-2027	STEC	O104:H4	ST678	+	+	-	+	+	-	-	-	AA	positive
EDL933	STEC	O157:H7	ST11	+	-	+	-	-	-	-	+	LA	negative
17-2	EAEC	O3:H2	ST10	-	+	-	+	+	+	-	-	AA	negative
E2348/69	EPEC	O127:H6	ST15	-	+	-	-	-	-	+	+	LA	negative

ST: Sequence Type; STEC: Shiga toxin producing *E. coli*; EAEC: Enteroaggregative *E. coli*; EPEC: Enteropathogenic *E. coli*; AA: aggregative adhesion; LA: localized adhesion

### Faecal screening study

On June 6^th^, 2011, faecal samples were taken from the gut of a total of 100 cattle, slaughtered on that day in one abattoir. The animals originated from 34 different farms in Northern Germany, the centre of the 2011 HUS outbreak. After diluting the samples in a ratio of 1:2 with PBS and vortexing, the samples were streaked on (i) CHROMagar Orientation (Mast Diagnostics, Paris, France), (ii) CHROMagar supplemented with cefotaxime (4 μg/ml) and (iii) Gassner agar plates (Oxoid GmbH, Wesel, Germany). After 18 h of incubation at 37°C a total of 20 coliform colonies per animal were picked from these three plates based on colony morphology and streaked on blood agar, resulting in the total number of 2.000 coliform colonies that were further investigated. The next day, the colonies were grouped into 4 pools per animal, each pool containing material of five coliform colonies and after heat lysis treatment (10 min, 100°C) the pools were analysed using three different Multiplex-PCRs (see below). If any gene detected in these Multiplex-PCRs yielded a positive result, the PCR was repeated with each single colony of the pool. Positive colonies were puri-fied and tested biochemically to confirm that these were *E. coli *after which each colony was now referred to as a bacterial strain.

### Determination of extended-spectrum beta-lactamase (ESBL)-producing *E. coli*

Colonies showing growth and the typical phenotype of *E. coli *on CHROMagar agar plates supplemented with cefotaxime were also tested bio-chemically to confirm they were *E. coli*, and ESBL-production was confirmed according to the CLSI criteria [26]. Colonies with a positive confirmatory test were further processed and regarded as bacterial strains. DNA was isolated using standard methods and used for PCR based screening and, if positive, subsequent sequencing of the ESBL related genes *bla*_CTX-M_, *bla*_TEM _and *bla*_SHV _[27].

### Determination of virulence-associated genes

Two of the three Multiplex-PCRs utilized in this study have been published previously. In brief, for the detection and identification of the O104:H4 outbreak strain, we used the recently described Multiplex-PCR for *stx2*, *terD*, *rfb*_O104_, and *fliC*_H4 _[3].

In a second part of the study, STEC and enteropathogenic *E. coli *(EPEC) were identified using a Multiplex-PCR including *stx1 *and *stx2 *(STEC) as well as *escV *and *bfpA *(EPEC) [28]. The screening for EAEC was performed by the detection of *pAA*, *aggR*, and *astA *by a Multiplex-PCR based on published primer sequences and single PCR protocols, respectively. Briefly, primers for the detection of the virulence-associated factors *pAA *(5'-CTGGCGAAAGACTGTATCAT-3' and 5'-CAATGTATAGAAATCCGCTGTT-3') [29], *aggR *(5'-GTATACACAAAAGAAGGAAGC-3' and 5'-ACAGAATCGTCAGCATCAGC-3') [30], as well as *astA *(5'-TGCCATCAACACAGTATATCC-3' and 5'-TAGGATCCTCAGGTCGCGAGTGACGGC-3') [31], were chosen according to primer compatibility and product size to fit into a single Multiplex-PCR. In brief, this Multiplex-PCR was performed in a 25 μl reaction mixture including 2.5 μl 10 × PCR buffer, 2.0 μl 50 mM MgCl_2_, 2U Taq DNA polymerase (Rapidozym, Germany), 0.5 μl of each 10 mM dNTP, 0.1 μl (100 pmol) oligonucleotide primer pair, and 4 μl template DNA, supplemented with the appropriate volumes of double-distilled water. Reaction mixtures were subjected to the following temperature profiles: 5 min at 94°C; 25 cycles of 30 s at 94°C, 1 min at 56°C, 45 s at 72°C, with a final circle of 10 min at 72°C and a hold at 10°C.

### Multi locus sequence typing (MLST) and phylogenetic grouping

ST678, the ST of the current HUS outbreak strain, according to the scheme described by Wirth et al. (2006) [32] (http://mlst.ucc.ie/mlst/dbs/Ecoli) can easily be identified by the analyses of two housekeeping genes only, namely *icd *and *mdh*, as the combination of *icd *allele 136 and *mdh *allele 9 specifically identifies this ST. Therefore, all identified STEC, phenotypic ESBL-producing *E. coli *and other isolates that harboured at least some of the genetic features common to the outbreak strain were further analysed by sequencing these two alleles.

All sequences generated for these two genes were concatenated and the phylogenetic relationship was inferred using the Maximum Parsimony method. Phylogenetic analyses were performed with Mega 5.05 (http://www.megasoftware.net) and CLC Genomics Workbench 4.7 (http://www.clcbio.com). Based on these analyses, complete MLST was performed for all strains that seemed to be very closely related to ST678. MLST determination was carried out as described previously [32] with minor modifications as published by Ewers et al. [27].

### Cell adhesion assay

As EAEC are a highly heterogeneous group of pathogens, we further tested selected strains (n = 27) for their adhesion pattern in HEp-2 cells, the gold standard for EAEC typing [12,33]. The selection of strains was based on the results obtained in the Multiplex-PCRs (particularly, but not exclusively, the ability to produce Shiga toxin), and/or close relation to the outbreak strain according to the MLST analysis (additional file 2). Briefly, HEp-2 cells were seeded in 12-well-plates with cover slips 48 hours before infection. After a three hour infection period using bacterial strains suspended in cell culture medium containing 1% mannose, HEp-2-cells on the cover slips were washed with 1× PBS three times, fixated with methanol for 15 minutes and stained with freshly prepared GIEMSA staining solution (45 minutes, room temperature) [13].

## Competing interests

The authors declare that they have no competing interests.

## Authors' contributions

Conceived and designed the study: LHW, AB, SG; Performed the experiments: AB, IE, BK, SG, EMA; Analyzed the data: LHW, TS, AB, LG; Wrote the paper: LHW, TS, AB, SG; Critically read the manuscript: HK, LG, EMA; Read and approved the final manuscript: LHW, TS, IE, EMA, BK, LG, HK, SG, AB

## Supplementary Material

Additional file 1 **Maximum Parsimony based clustering analysis of the concatenated sequences of the 7 genes used for MLST. Reference scale of tree is equal to 1 nucleotide substitution**. Maximum Parsimony based clustering analysis of the concatenated sequences of the 7 genes used for MLST. Reference scale of tree is equal to 1 nucleotide substitution. Click here for file

Additional file 2 **Characteristics of strains selected for cell adhesion assay (n = 27) and reference strains**. Click here for file
