# Supplementary figures and images for: No evidence of the Shiga toxin-producing E. coli O104:H4 outbreak strain or enteroaggregative E. coli (EAEC) found in cattle faeces in northern Germany, the hotspot of the 2011 HUS outbreak area
Source: Gut Pathog. 2011 Nov 3;3:17. doi: 10.1186/1757-4749-3-17 (PMC3227623; doi:10.1186/1757-4749-3-17)

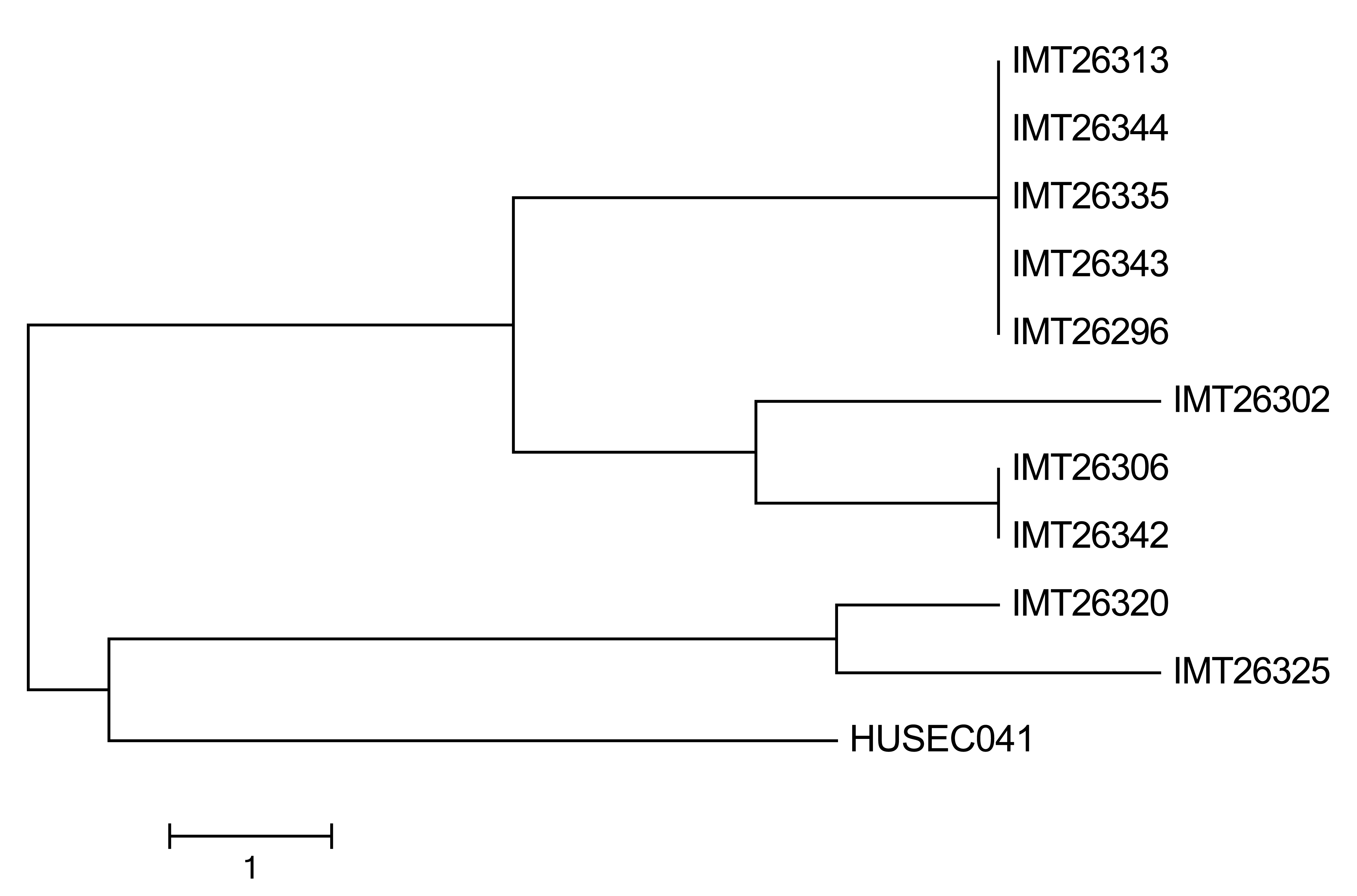

Supplement: Additional file 1 — Maximum Parsimony based clustering analysis of the concatenated sequences of the 7 genes used for MLST. Reference scale of tree is equal to 1 nucleotide substitution. Maximum Parsimony based clustering analysis of the concatenated sequences of the 7 genes used for MLST. Reference scale of tree is equal to 1 nucleotide substitution. [file 1757-4749-3-17-S1.TIFF]
