# Supplementary material for: No evidence of the Shiga toxin-producing E. coli O104:H4 outbreak strain or enteroaggregative E. coli (EAEC) found in cattle faeces in northern Germany, the hotspot of the 2011 HUS outbreak area
Source: Gut Pathog. 2011 Nov 3;3:17. doi: 10.1186/1757-4749-3-17 (PMC3227623; doi:10.1186/1757-4749-3-17)
Supplement: Additional file 2 — Characteristics of strains selected for cell adhesion assay (n = 27) and reference strains. [file 1757-4749-3-17-S2.DOC]

Additional file 2: Characteristics of strains selected for cell adhesion assay (n=27) and reference strains

| **Strain** | **Animal-no.** | **Farm-no.** | **MLST** | | **VAG** | | | | | | | | | | **Adhesion pattern** |
| --- | --- | --- | --- | --- | --- | --- | --- | --- | --- | --- | --- | --- | --- | --- | --- |
|  |  |  | *icd*** | *mdh*** | *stx2* | *stx1* | *terD* | *rfb_O104_* | *fliC_H4_* | *pAA* | *aggR* | *astA* | *bfpA* | *escV* |  |
| IMT26296 | 16 | F6 | 26 | 9 | + | - | - | - | - | - | - | - | - | - | No defined pattern |
| IMT26299 | 28 | F16 | 1 | 9 | + | + | - | - | - | - | - | - | - | - | LA |
| IMT26300 | 28 | F16 | 1 | 9 | + | + | - | - | - | - | - | - | - | - | LA |
| IMT26302 | 32 | F17 | 18 | 9 | - | - | - | - | + | - | - | - | - | - | LA |
| IMT26305 | 42 | F18 | 26 | 9 | + | + | - | - | - | - | - | - | - | - | No defined pattern |
| IMT26306 | 42 | F18 | 26 | 11 | - | - | + | - | - | - | - | - | - | - | No defined pattern |
| IMT26308 | 43 | F18 | 109 | 7 | + | - | - | - | - | - | - | + | - | - | No defined pattern |
| IMT26313 | 51 | F20 | 26 | 9 | + | + | - | - | - | - | - | - | - | - | No defined pattern |
| IMT26315 | 57 | F20 | new | new | + | - | - | - | - | - | - | + | - | - | LA |
| IMT26316 | 60 | F20 | new | 9 | + | - | - | - | - | - | - | - | - | - | No adhesion detected |
| IMT26318 | 68 | F22 | 8 | 8 | + | - | - | - | + | - | - | + | - | - | No defined pattern |
| IMT26319 | 71 | F24 | 18 | 53 | + | - | + | - | - | - | - | + | - | - | No defined pattern |
| IMT26320 | 73 | F25 | 18 | 9 | + | - | - | - | - | - | - | - | - | - | DA |
| IMT26322 | 74 | F26 | 1 | 9 | + | + | - | - | - | - | - | - | - | - | No defined pattern |
| IMT26323 | 74 | F26 | 8 | 8 | - | - | - | - | - | - | - | - | - | - | LA |
| IMT26325 | 74 | F26 | 18 | 9 | + | - | - | - | - | - | - | - | - | - | No defined pattern |
| IMT26330 | 75 | F34 | 16 | 24 | + | - | - | - | - | - | - | - | - | - | No defined pattern |
| IMT26333 | 80 | F10 | 16 | 12 | - | - | - | - | - | - | - | - | - | - | LA |
| IMT26335 | 83 | F10 | 26 | 9 | + | + | - | - | - | - | - | - | - | - | No defined pattern |
| IMT26342 | 92 | F10 | 26 | 9 | + | - | - | - | - | - | - | - | - | - | No defined pattern |
| IMT26343 | 93 | F10 | 26 | 9 | + | + | - | - | - | - | - | - | - | - | No defined pattern |
| IMT26344 | 93 | F10 | 26 | 9 | + | + | - | - | - | - | - | - | - | - | No defined pattern |
| IMT26368 | 36 | F18 | 13 | 36 | - | - | - | - | + | - | - | - | - | - | No defined pattern |
| IMT26406 | 69 | F22 | n.t. | n.t. | - | - | - | - | - | - | - | - | - | + | LA |
| IMT26407 | 69 | F22 | n.t. | n.t. | - | - | - | - | + | - | - | - | - | - | No defined pattern |
| IMT26408 | 86 | F10 | n.t. | n.t. | - | - | + | - | - | - | - | - | - | + | LA |
| IMT26409 | 82 | F10 | n.t. | n.t. | - | - | - | - | + | - | - | - | - | - | No defined pattern |
| 17-2 | none | none | 8 | 8 | - | - | + | - | - | + | + | + | - | - | AA |
| 2348/69 | none | none | 15 | 18 | - | - | + | - | - | - | - | - | + | + | LA |
| RKI II-2027 | none | none | 136 | 9 | + | - | + | + | + | + | + | - | - | - | AA |

*: n.t.: not tested; **: new: allele not yet assigned ([www.mlst.net](http://www.mlst.net/)); LA: localized adherence; DA: diffuse adherence; AA: aggregative adherence
